# A systematic review of evidence that enteroviruses may be zoonotic

**DOI:** 10.1038/s41426-018-0159-1

**Published:** 2018-09-26

**Authors:** Jane K. Fieldhouse, Xinye Wang, Kerry A. Mallinson, Rick W. Tsao, Gregory C. Gray

**Affiliations:** 1 Division of Infectious Disease and Global Health Institute, Duke University, Durham, NC 27710 USA; 2 Global Health Research Center, Duke Kunshan University, Kunshan, 215316 Jiangsu China; 3 Program in Emerging Infectious Diseases, Duke-National University of Singapore Medical School, Singapore, 169857 Singapore

## Abstract

Enteroviruses infect millions of humans annually worldwide, primarily infants and children. With a high mutation rate and frequent recombination, enteroviruses are noted to evolve and change over time. Given the evidence that human enteroviruses are commonly found in other mammalian species and that some human and animal enteroviruses are genetically similar, it is possible that enzootic enteroviruses may also be infecting human populations. We conducted a systematic review of the English and Chinese literature published between 2007 and 2017 to examine evidence that enteroviruses may be zoonotic. Of the 2704 articles screened for inclusion, 16 articles were included in the final review. The review of these articles yielded considerable molecular evidence of zooanthroponosis transmission, particularly among non-human primates. While there were more limited instances of anthropozoonosis transmission, the available data support the biological plausibility of cross-species transmission and the need to conduct periodic surveillance at the human–animal interface.

## Introduction

Enteroviruses (EVs) are positive-sense, single-stranded RNA viruses in the family *Picornaviridae* that infect millions of people worldwide on an annual basis, especially infants and children under the age of one^1^. More than 300 serotypes of EV have been identified and demonstrated to cause a variety of diseases and morbidity^2^. Among the most notable EVs known to infect humans are coxsackie-, echo-, polio-, and rhinoviruses. While a majority of non-polio EV infections are asymptomatic or cause mild respiratory disease, more severe disease outcomes such as aseptic meningitis, acute flaccid paralysis (AFP), and acute hemorrhagic conjunctivitis are associated with certain EV types. Enterovirus 71 (EV-71), for example, is a type most commonly associated with severe hand, foot, and mouth (HFMD) disease. Enterovirus D68 (EV-D68) is a type that has caused sporadic but severe respiratory disease outbreaks across the United States, Asia, Africa, and Europe in recent years^3,4^.

In 2012, a comparative analysis of phylogenic and genetic classification demonstrated a close relationship between animal and human picornaviruses^5^. In February 2013, the International Committee on Taxonomy of Viruses (ICTV) approved changes to EV and rhinovirus species names to remove all reference to the host species names. Former species names had been selected based upon the host species from which the virus was originally isolated; however, after many of the human EV species were identified and isolated in non-human hosts, the proposal was made to drop the designation of the viruses as human, bovine, porcine, and simian. Today, EVs are classified into 12 species, including enterovirus A–L (EV-A, B, C, D, E, F, G, H, J, K, and L) and rhinovirus A–C (RV-A, B, and C) (Table 1). EVs E–G chiefly cause disease in livestock, such as cattle^6^ and pigs^7^, while several simian EVs (e.g., EV-H and EV-J) have been isolated from both captive and wild non-human primates (NHPs)^2,8^. Enterovirus I has historically been excluded for fear of confusion with enterovirus 1; however, a dromedary camel enterovirus was proposed as a new species of EV I to the ICTV in June 2016^9^.

**Table 1:** Species within the *Enterovirus* genus

Current species name	Former species name	Number of unique viruses recognized
*Enterovirus* A	*Human enterovirus* A	25
*Enterovirus* B	*Human enterovirus* B	63
*Enterovirus* C	*Human enterovirus* C	23
*Enterovirus* D	*Human enterovirus* D	5
*Enterovirus* E	*Bovine enterovirus* (group A)	4
*Enterovirus* F	*Bovine enterovirus* (group B)	6
*Enterovirus* G	*Porcine enterovirus* B	20
*Enterovirus* H	*Simian enterovirus* A	1
*Enterovirus* I	–	1
*Enterovirus* J	Unclassified simian viruses	6
*Enterovirus* K	–	1
*Enterovirus* L	–	1
*Rhinovirus* A	*Human rhinovirus* A	80
*Rhinovirus* B	*Human rhinovirus* B	32
*Rhinovirus* C	*Human rhinovirus* C	56

Derived from: refs. ^51,52^

Given the evidence that human EVs may be commonly found in other mammalian species, it is possible EVs that naturally circulate in animal populations may also be infecting human populations. With multiple genotypes, a high mutation rate, and frequent recombination^10^, EVs have considerable potential for cross-species infection. Recognizing that the term “zoonoses” can be confusing^11^, for the purposes of this review, we defined the generic term “zoonoses” as a “two-way street” where a pathogen causing disease might move from either animals to humans or from humans to animals^12,13^. Where we sought to be directionally more specific, we employed the term “anthropozoonosis”, which we embraced as “a disease causing pathogen that is transmitted from animals to humans”, and “zooanthroponosis”, sometimes referred to as “reverse zoonoses”, as “a disease causing pathogen that is transmitted from humans to animals.” Therefore, in this report, we sought to review the English and Chinese scientific literature for evidence that EVs may be zoonotic.

## Methods

In August 2017, we searched the English literature published between 2007 and 2017 on ProQuest, PubMed, Scopus, and Web of Science databases. The systematic review of Chinese literature published between 2007 and 2017 was conducted in September 2017 on the Chinese National Knowledge Infrastructure (CNKI), Wanfang Data and Weipu Data databases. Both reviews followed the standard systematic review procedures established by the Preferred Reporting Items for Systematic Reviews and Meta-Analyses (PRISMA). In each of these databases, we empirically chose to search for citations during the last 10 years as molecular methods for virus detections have markedly increased during the last decade as compared to older diagnostic methods such as cell culture and immunoassays. The search was additionally limited to include the following publication categories: review, article, dissertation, thesis, or journal. Articles were included in the search if they mentioned “enterovirus”, “rhinovirus”, a virus that belongs to the EV genus (“coxsackie”, “echovirus”, “poliovirus”, “pleurodynia”, “simian virus 6”, “SV6”, and “unclassified simian virus”), or a disease caused by an EV (“Hand, foot, and mouth disease”, “Bornholm disease”, and “Swine vesicular disease”) as well as a term of interest (“human infection”, “transmiss*”, “transfer”, “cross-species”, “interspecies”, “zoono*”, “anthropono*”, or “zooanthropono*”) in either the title, abstract, or keywords. The search methods were designed to capture any documentation of zoonotic transmission, regardless of the terms used to describe the type of transmission.

The following search string was used for the English databases: (enterovirus* OR rhinovirus* OR coxsackie* OR echovirus* OR poliovirus* OR “Hand, foot, and mouth disease” OR “Bornholm disease” OR pleurodynia OR “Simian virus 6” OR SV6 OR “unclassified simian virus*” OR “Swine vesicular disease”) AND (“human infection” OR transmiss* OR transfer OR cross-species OR interspecies OR zoono* OR anthropono* OR zooanthropono*). Articles published in languages other than English were not considered during the English literature review.

Concurrently with the English literature review, we conducted a systematic review of the Chinese literature due to the high annual disease burden of EVs in China. Between 2008 and 2013 alone, China experienced epidemic outbreaks and saw over 9 million reported cases of HFMD caused predominantly by EV serotypes A71 (EV-A71) and Coxsackievirus A16 (CV-A16)^14,15^. From a One Health perspective, China is also seeing increasingly frequent and intense contact between humans and domestic livestock largely due to the rapid increase in demand for meat, poultry, and dairy products. Furthermore, habitat loss and illegal wildlife trade have recently increased human exposure to wild animals^16^. Conceivably, Chinese populations may be more likely to experience animal EV infections than humans in other societies.

The search terms used for the Chinese literature review were based on the English search terms: (SU =  ‘enterovirus (Changbingdu)’ OR SU = ‘coxsackie (Kesaqibingdu)’ OR SU = ‘echovirus (Aikebingdu)’ OR SU = ‘rhinovirus (Bibingdu)’ OR SU = ‘poliovirus (Xiaoermabizheng)’ OR SU = ‘hand, foot, and mouth disease (Shouzukoubing)’ OR SU = ‘bornholm disease (Liuxingxingxiongjitong)’ OR SU = ‘pleurodynia (Xiongmotong)’ OR SU = ‘simian virus (Yuanhoubingdu)’ OR SU = ‘Swine vesicular disease (Zhushuipaobing)’) AND (SU = ‘zoonoses (Renchugonghuan)’ OR SU = ‘human infection (Renganran)’). All articles were in Chinese. The search terms “cross-species”, “anthropono*”, and zooanthropono*” were not included when searching in the Chinese databases as these terms greatly limited the number of articles generated. EndNote X8 was utilized to compile articles and remove duplicates for both the English and the Chinese literature reviews.

## Results

### Search results

**Fig. 1:**
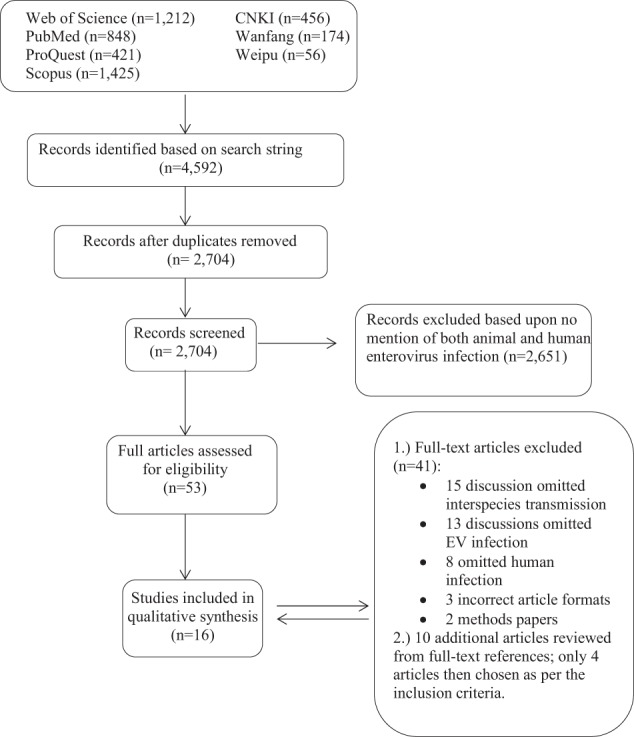
Flow chart of the literature search process. Based on the search strategy, 4592 articles were identified in total, which included 3906 English articles and 686 Chinese articles. Duplicates were removed

A total of 4592 articles were identified by our search strategy. After duplicates were removed, a total of 2704 articles were screened for inclusion (Fig. 1). The title, abstract, and keywords of all English articles captured by the search were independently reviewed by two authors (J.K.F. and R.W.T. reviewed articles by authors A-L; X.W. and K.A.M. reviewed articles by authors M-Z). The abstracts of 686 Chinese articles were reviewed by X.W. Articles that made no mention of both animal and human infection of an EV were not considered for full-text review. Following the screening of both English and Chinese articles, 53 English articles were selected for full-text review, which was conducted independently by each author for inclusion. After the full review, 41 articles were removed due to omission of interspecies transmission, omission of EV infection, omission of human infection, incorrect article format or the article being a methods paper (Fig. 1). From the 12 articles selected for final inclusion in the qualitative synthesis, an additional ten articles were identified from the references as potentially significant and were, therefore, reviewed in full. Four of the ten additional articles met the inclusion criteria; thus, a total of 16 English publications were finally chosen in this review (Table 2).

**Table 2:** Publications found to be important in considering the zoonotic potential of enteroviruses

Publications	Country and year	Main summary	Strength of evidence
Gür et al.^17^	Turkey 2008	Bovine enterovirus type 1-specific antibodies were detected using a microneutralization test in sera from 74 out of 244 humans living in urban areas of Turkey (no report of clinical infection), as well as horses, dogs, goats, and sheep.	Serological study; humans sampled reportedly healthy
Oberste et al.^30^	USA 2008	Genome sequences of simian enteroviruses SV6, SV19, SV46, and enteroviruses EV92 and EV103 detected in captive primates demonstrated close phylogenetic relationships to HEV-A. The EV103-POo-1 amino acid sequences also shared 93 and 96% identity with the SV6 on the P2 and P3 regions.	RT-PCR and complete genome sequencing
Smura et al.^34^	Finland 2011	A review of enterovirus species evolution includes a description of possible zoonotic origin of EV-70.	Review article
Harvala et al.^23^	Cameroon 2011	Enteroviruses infecting wild chimpanzees were characterized and found to be related to human strains detected in patients in Central Africa, including LM1677 (clustered closely with EV79 in VP1 and VP4/VP2 regions) and KK2640 (grouped closely with the EV70 in the VP1 region and with the EV94 in the 5′UTR and 3Dpol).	RT-PCR and near-complete genome sequencing
Nielsen et al.^31^	Denmark 2012	A near-complete viral genome of a human strain of coxsackie B3 (CB3) virus was found to be the cause of severe respiratory symptoms and death in a chimpanzee at a zoo in Copenhagen.	RT-qPCR and near-complete genome sequencing
Oberste et al.^32^	Bangladesh 2013	Among non-human primates at a zoo in Dhaka, 12.5% of all enteroviruses detected (8/64) based on an analysis of the VP1 region were EVs previously detected in humans. The most common human enterovirus was echovirus 24 (E24). These results are surprising considering the authors’ findings among synanthropic NHP populations (Oberste et al.^33^).	RT-qPCR and partial genome sequencing
Oberste et al.^33^	Bangladesh 2013	Twenty human EVs were detected in synanthropic NHP in Bangladesh with four from HEV-A, 13 from HEV-B and three from HEV-C types determined by the partial VP1 sequencing.	RT-qPCR and partial genome sequencing
Sadeuh-Mba et al.^24^	Cameroon 2013	The strain C08-142 was isolated from a patient with AFP; this strain is related to EV-A76 strain LM1677 and was previously detected in a wild chimpanzee in Cameroon. Similarly, EV-D111 was isolated from healthy child; this strain is closely related to the KK2640, which was also isolated from chimpanzee in Cameroon.	Viral isolation, RT-PCR, and partial genome sequencing
Harvala et al.^26^	Cameroon, DRC 2014	Species A enteroviruses EV-A76, EV-A89 (based on the VP4 sequences), and A119 (based on the VP4 and VP1 sequences) were detected in apes in Cameroon. EV-A76 and EV-A89 were also isolated from human stool specimens from patients with AFP in a previous study (Oberste et al.^29^).	RT-PCR, viral isolation and partial genome sequencing
Sadeuh-Mba et al.^25^	Cameroon 2014	Coxsackievirus A13 and A24, Echovirus 15 and 29 and EV-B82 (VP1 region) were detected in stool samples of captive chimpanzees and gorillas. In addition, EV-A76 (wildly circulated in humans) was found in wild chimpanzees.	RT-snPCR, partial genome sequencing and viral isolation
Mombo et al.^27^	Congo 2015	First identification of a human EV-C (EV-C 99; targeting the capsid gene VP1) found to be associated with AFP in a chimpanzee.	RT-qPCR, and near-complete genome sequencing
Bruhn et al.^36^	USA 2015	Through recombination analysis and phylogenic analysis of 51 SVDV samples (27 from this study and 24 from a previous study) provide evidence that SVDV originates from a single recombinant origin of CV-B5 and CV-A9, supporting the hypothesis of a single anthroponotic transfer origin (human to pig).	RT-PCR and near-complete genome sequencing
Grützmacher et al.^19^	CAR 2016	A gorilla fecal sample tested positive for EV most similar to simian agent 5 B165; a human fecal sample tested positive for EV most similar to swine vesicular virus (89% identity, 5′UTR; Accession: AY875991.1) and human enterovirus 71 (90% identity, partial, gene for polyprotein, Accession: AB575924.1).	RT-qPCR and partial genome sequencing
Du et al.^22^	China 2016	Sequencing of EV from rodents in china detected 72% identity with human coxsackie virus A11 (nt sequence 295-632 of 5′UTR) and 73% identity with EV-D68 (179-628 of 5′UTR).	Sequence-independent PCR and next-generation sequencing
Lomakina et al.^37^	Russia 2016	Coxsackie B4 characterization demonstrated phylogenetic evidence that SVDV emerged from a human ancestor between 1945 and 1975 (T75 diverging from human CVB4 after 1945).	Viral isolation, Sanger sequencing using PCR
Mombo et al.^28^	Gabon 2017	Thirty-two out of 600 fecal samples from wild apes and monkeys were positive for EVs with HEV-A and HEV-B strains (targeting the VP1 and VP2 regions) detected in the chimpanzee samples and HEV-B and simian EV-J identified in mandrill samples.	RT-qPCR and partial genome sequencing

*RT-PCR* reverse transcription-polymerase chain reaction, *RT-qPCR* real-time reverse transcription-polymerase chain reaction, *RT-snPCR* semi-nested reverse transcription-polymerase chain reaction, *HEV-A* human enterovirus A, *NHP* non-human primate, *AFP* acute flaccid paralysis, *SVDV* swine vesicular disease virus

Five of the 16 manuscripts included in the review were published prior to 2013 when the ICTV approved changes to EV species names. The 16 selected publications described studies conducted across 11 distinct countries with 43% of the studies (*n* = 7) clustered in Central African countries, 25% (*n* = 4) in Europe, 12% (*n* = 2) in the United States, and 19% (*n* = 3) in Asia. Eleven of the 16 studies (69%) discussed the zooanthroponoses of EVs (primarily among NHPs) and two of the articles (13%) discussed possible evidence of anthropozoonoses; however, through phylogenetic or molecular evidence, all 16 articles included in the final analysis documented instances of animal EV infection in humans or human EV infection in animals.

### Evidence of anthropozoonosis

Two of the studies included in the review documented possible evidence of anthropozoonotic infection of humans with animal EVs^17^. The 2008 Gür et al.^17^ study collected a total of 3020 serum samples from eight species in different regions of Turkey. A microneutralization test provided serological evidence of bovine enterovirus type 1 (BEV-1) in 74 out of 244 healthy adults (30.3%) in Konya, Turkey. After cattle (64.8%), humans had the highest ratio of seropositivity for BEV-1 compared to four other species that tested positive, including sheep (32.8%), goats (27.6%), horses (12.8%), and dogs (3.2%). As a serological study, this article could not provide evidence of active infection or transmission route; however, based on the previous evidence^18^, the authors suggest the possible cause of human BEV-1 infection could be via contact with infected animals and/or contaminated cattle feces. All samples came from reportedly healthy adults living in urban areas, where contact with infected cattle feces was presumably lower than in rural areas. During this systematic review we found no other publication that documented seropositivity for BEV-1 in humans subsequent to this 2008 study.

Grützmacher et al. reported several respiratory disease outbreaks occurring simultaneously among gorilla populations and humans residing in nearby research camps in the Dzanga Sangha Protected Areas of the Central African Republic^19^. The study noted that respiratory symptoms manifested in the human population prior to the first respiratory signs being noticed among the gorilla population. In the first outbreak investigated, one of the 16 human specimens (fecal samples and throat swaps) collected tested positive for EV by PCR and viral sequencing. When analyzed using the National Center for Biotechnology Information’s (NCBI) Basic Local Alignment Search Tool (BLAST), the human specimen most similarly matched swine vesicular virus (isolate ITL 2/92 5′ UTR; Accession: AY875991.1) and human EV-71 (isolate 17001, gene for polyprotein, partial cds; Accession: AB575924.1), with 89 and 90% similar identity, respectively. Additionally, one of the seven fecal samples tested positive for an EV with 90% identity to Simian agent 5 (strain B165, complete genome; Accession: AF326751.2) based on the NCBI database. In the third outbreak, 12 out of 25 fecal samples collected from gorillas were positive for EV. Interestingly, sequences of these positive samples were identical with the human EV sequence detected in the first outbreak, which also matched with a high percentage both swine vesicular disease virus (SVDV) and EV-A 71. SVDV is believed to be evolved from coxsackie B virus serotype 5 (CBV-5) and is generally regarded as an animal EV^20,21^. Although this study does not provide evidence of how humans were infected with animal EVs, these results still demonstrate the possible zoonotic potential of EVs.

### Evidence of zooanthroponosis

As compared to the evidence of anthropozoonotic EV transfer from animals to humans, we found that the majority of publications included in the final review (*n* = 11) demonstrated evidence of zooanthroponosis EV transfer from humans to animals. Ten of these publications documented evidence of human EV infections in NHPs, specifically chimpanzees, gorillas, mandrills, and Old-World monkeys (OWMs) such as macaques and baboons. One publication provided evidence that human EVs might also infect rodents^22^.

Interestingly, six of these 11 publications were from studies clustered in Central Africa: three cited studies of NHP EV-infections only in Cameroon^23–25^, one in both Cameroon and the Democratic Republic of the Congo (DRC)^26^, one in the DRC^27^, and one in Gabon^28^.

Harvala et al. screened chimpanzee and gorilla stool samples in the jungles of Cameroon where there was reportedly very minimal human contact^23^. The study found chimpanzee EVs that closely related to human EV-A and -D species in the complete VP1 region, including EV-A76 strains and a new species D type, assigned as EV-D111. Although it is unclear if the EVs detected in these fecal samples were indigenous to the chimpanzee populations or were directly or indirectly transmitted from humans, scholars still provide evidence about the possible cross-species transmission of EVs between humans and NHPs in Cameroon. Subsequently, in their 2014 publication, Harvala et al.^26^ similarly detected human EVs in stool samples from chimpanzees, gorillas, and bonobos in other forested areas of Cameroon and the DRC, including EV-A76. In addition, the VP4 sequences of viruses isolated from chimpanzees had close identity to EV-A89 and a newly identified species A type (EV-A119) was detected from chimpanzees and gorilla in this study (both VP4 and VP1 sequences). These two types of EVs that have been demonstrated to infect humans^24,25,29^. Citing the Harvala et al. studies^23,26^, Sadeuh-Mba et al. found similar results when characterizing stool specimens collected from both captive and wild NHPs, with 18 out of 21 EVs (including EV-A76, EV-A71, Echovirus-15, Echovirus-29, EV-B82, Coxsackievirus-A13, and Coxsackievirus-A24) detected in chimpanzees and gorillas and one of six monkey-derived EVs reportedly circulating in human populations^25^. In their previous study of human EVs circulating in both healthy children and patients with AFP, Sadeuh-Mba et al. isolated several viruses that had been detected in wild chimpanzees, including EV-D111 and EV-A76^24^. Using viral isolation, RT-PCR and sequencing, the 2014 Sadeuh-Mba et al. publication also provides strong evidence to support the hypothesis that the diversity of EVs in NHPs might be much broader than previously understood in the Harvala et al. studies^23,26^. For example, two new EVs, sharing only 55.6–57.9% nucleotide and 52.2–53.2% amino acid VP1 sequence identity with the closest EV (SV6), were identified during this study and proposed as new EV types EV-122 and EV-123.

Mombo et al. was the first report to document that a human EV-C99 was associated with AFP symptoms in a captive chimpanzee in Congo^27^. Two years later, Mombo et al. screened fecal samples from wild-living primates (include OWMs and apes) and through nucleic acid detection detected a novel human EV-B type (EV-B11) in mandrills, as well as human EV-A type (closely related to EV90) and EV-B type (EV107 and EV-B112) in chimpanzees^28^. These findings are all consistent with previously published reports in this review.

Studies across the US^30^, Europe^31^, and Asia^32,33^ supported the findings of human EV infection in NHPs, predominantly among captive primates. Interestingly, Oberste et al. found that nearly all EVs detected in synanthropic NHPs in Dhaka, Bangladesh, were human EVs based on VP1 sequencing, whereas only 12.5% of the detected EVs among NHPs in a zoo in Dhaka were human EVs^32,33^. A 2016 study in China provided some of the only evidence of EV infection among species other than NHPs. The EVs detected in rodents shared 72% identity with human coxsackievirus A11 based on the nucleotide sequence from 295-632 of the 5′UTR (Tibet2015) region and 74% identity with EV-D68 based on 179-628 of 5′UTR (NX2015) region^22^.

### Supporting phylogenic evidence

It is well documented that SVDV originated in humans as human coxsackie virus B5, which transferred to pigs between 1945 and 1965^34,35^. Through recombination and phylogenetic analysis, Bruhn et al. recently suggested that SVDV may have originated from a single recombinant event^36^. More recently, a virus isolate (T75) from a 1975 SVDV outbreak in Central Russia was propagated, sequenced, and compared to an isolate from the first reported outbreak of SVDV in the Soviet Union (strain O72). Lomakina et al. found the T75 strain shared 80.0–90.4% identity with human coxsackievirus B4 in the VP1 region using Sanger sequencing. However, unlike the CVB5-related SVDV, which has caused several epizootic outbreaks since the 1960s, T75 has not become an established swine pathogen^37^. Our understanding that SVDV originated as a human EV supports the finding that cross-species transfer of EVs occurs in animal species beyond NHPs.

## Discussion

The majority of the studies included in this review clearly documented that animal infection with several HEV types (including EV-A, B, C, and D) have at least occasionally occurred. Interestingly, among these studies, the reverse zoonoses of EVs between NHP populations and humans are discussed most frequently. It also became evident through the review that there have been a number of diverse HEVs identified in NHPs. For instance, the Harvala et al. studies^23,26^ and Sadeuh-Mba et al. studies^24,25^ detected diverse EV species, such as species A (A76, A89, A90, A119), species B (B110), and species D (D111, D120) through screening of fecal samples collected from wild chimpanzees and gorilla in Central African countries (primarily in Cameroon). These EV types have been also detected in humans or were found to be closely related to previously detected HEV types. Similarly, the Oberste et al. studies conducted in Bangladesh also reported that several types of HEVs (including species A, B, and C) detected from both captive and wild NHPs (primarily Rhesus macaques)^32,33^. It is worth noting that EV-A76 was detected from both human samples and urban rhesus macaques in Bangladesh as well, although EV-A76 was not the most prevalent picornavirus detected among NHPs in the study. In similar studies conducted in Denmark and the USA, although researchers did not find the same types of HEVs as previously described in the studies conducted in Bangladesh and Central Africa, authors did find several new types of EVs that also closely related to HEVs circulating in rhesus macaques, including Coxsackie B3 (CB3, now assigned into species B)^31^, simian enterovirus 46 (SV46), and EV92^30^. These results may indicate that NHPs are susceptible to more diverse types of HEVs than previously expected.

In contrast to human infections with HEVs, which have been widely studied in the past decades, NHP infections with HEVs have not historically received as much attention. Based on the evidence from a growing number reported instances of NHP infection with diverse HEVs, as well as several instances of human infection with non-human EVs, there are compelling data to show the possibility for cross-species EV transmission^23–26,32,33,38^. It is, therefore, important to understand the relationship between humans and NHPs and consider the susceptibility of NHPs to HEV infection.

Numerous previous reports mentioned that NHPs shared more than 90% of human DNA. Among these NHPs, chimpanzees (>99% with human DNA) were reported to be humans’ closest living relatives^38,39^. Our findings in this review support the hypothesis that NHPs (especially chimpanzees) may be at an increased risk for infection with human diseases because of their genetic similarity and due to less of a species barrier for pathogen transfer. However, the condition that this phenomenon is hinged on is close contact between NHPs and humans. It is well documented that there has been an increase in contact between NHPs and humans in recent decades, especially in Central African countries and South Asian countries^40^. For instance, Wolfe et al. and Betsem et al. describe how the growing demand for bushmeat and intensified deforestation of tropical forests in Central Africa have escalated humans’ hunting activities^41,42^. These hunting practices increase wild NHPs’ proximity to humans and heighten the risk of human pathogen infections in NHPs. In addition, due to rapid urbanization, an increase in population density and change in land use (i.e. forest encroachment) in recent decades, many South Asian countries (i.e. Singapore, and Bangladesh) have reported the NHPs (e.g. Rhesus macaques and long-tailed macaques) thrive in new human habitats, also thereby increasing the interactions between humans and NHPs^33,43^.

In 2007, Wolfe et al. reported, “primates constitute only 0.5% of all vertebrate species but have contributed about 20% of our major human diseases”^44^. Indeed, there are many human infectious diseases with NHP origins that are well-known and documented, including HIV/AIDS^45^, dengue^46^, hepatitis B^47^, and malaria^48^. Although no studies included in this review demonstrated human infection with an NHP EV, many of them indicate that NHP infection with human EVs might amplify human viruses or even facilitate recombination with primate EVs, yielding a novel source of future emerging diseases in humans. Thus, future studies exploring cross-species transmission of EVs between NHPs and humans are still needed.

The zoonotic transfer of EVs is not, however, limited to NHPs and humans; other animal EVs have also been found to infect humans and diverse animal species, such as BEV and SVDV. Subsequent to the Gür et al.^17^ study of BEV-1 antibodies in humans, horses, dogs, goats, and sheep, McClenahan et al. (2013) found that alpacas had been infected with BEVs in the United States^49^. The review also demonstrated the close relationship between SVDV and an HEV-B serotype, coxsackievirus B5 (CVB5), suggesting that SVDV was produced by the recombination between CVB5 and one other EV-B serotype^17,37^. These findings highlight the potential for cross-species transmission of SVDV. Cross-species transmission of animal EVs may also increase the risk for new emerging infectious diseases, as animal EVs may more easily mutate or recombine with other viruses in an animal host.

This systematic review had a number of limitations. First, as we limited the online review to studies published during the past ten years (focusing upon the more frequent use of molecular methods), the review may have missed some early reports of possible EV zoonoses. For example, we learned of the 1973 report documenting probable SVDV infections among workers at the Animal Virus Research Institute (United Kingdom) through serological assessment^21^ through an external reviewer of the manuscript. Similar, non-molecular evidence of zoonoses may have been missed. Second, through our search strategy we focused heavily upon zoonoses, and may have missed some important reports of cross-species infections among nonhuman animals^50^. We cannot exclude the possibility that partial genomes of viruses detected in animal feces were pathogens passing through the digestive system without active infection. Additionally, this review only found limited articles demonstrating evidence of anthropozoonosis  EV infection. Although the findings of these studies demonstrate the potential for animal EVs to infect humans, these results are not generalizable. For example, as a serological study, the Gür et al. study^17^ cannot provide evidence of active BEV-1 infection. In the other study demonstrating possible evidence of anthropozoonosis , Grutzmacher et al. found only one human fecal sample testing positive for an animal EV. While our findings demonstrate that EVs may be transmitted from animals to humans, the current scientific data are still somewhat sparse and provide suggestive, but not conclusive, evidence.

However, all of these findings convey an important message: the diversity of EVs and potential for cross-species transmission of EVs between humans and animals should not be underestimated. Further surveillance for cross-species EV infections among both humans and animals seem warranted. By leveraging a One Health approach, veterinarians, epidemiologists, and public health stakeholders will most effectively be able to provide targeted surveillance for EVs circulating in humans and animals. We recognize the barriers to active surveillance for EVs, including access to timely diagnostic tests, cross-reactivity in serology, and the limitations of primer-based molecular assays in detecting human as opposed to new animal EVs. As molecular diagnostic techniques continue to evolve, however, researchers may turn to more sensitive surveillance techniques, such as unbiased next-generation DNA sequencing as a solution to some of these hurdles.

## Conclusions

This review found considerable molecular evidence supporting the occurrence of zooanthroponoses of EV infection, particularly among NHPs. While it is more limited, serological evidence of BEV-1 in humans and phylogenic evidence of an SVDV-like infection in a human fecal sample, demonstrate the possibility of anthropozoonotic transfer. From a One Health perspective, we must consider that anthropogenic factors such as deforestation combined with an overall global increase in industrialized animal production has led to intensified and more frequent interactions between humans and animals. In particular, should contact between humans and NHPs increase the risk of zoonotic transfer, active surveillance for EV infections will be increasingly important due to deforestation and the resulting trend of escalated interactions between wild primate populations and humans. Given our understanding of the possibility for zoonotic transfer, and the knowledge that zooanthroponotic transfer of EVs may commonly occur, surveillance for cross-species transmission of enteroviruses is recommended, regardless of the directionality of the transfer.
